# *Mycobacterium bovis* infected domestic cats in an officially bovine tuberculosis free country resulting in human infection

**DOI:** 10.1016/j.onehlt.2025.101048

**Published:** 2025-04-21

**Authors:** Susanna Commandeur, Marleen van der Most, Jeroen Koomen, Lucien van Keulen, Annemieke Dinkla, Xander Luinenburg, Marieke Escher, Pieter Jacobs, Ingrid Keur, Guy C.M. Grinwis, Erik Weerts, Els M. Broens, Richard Anthony, Miranda Kamst-van Agterveld, Karin Rebel, Erik Huisman, Marloes Heijne, Ad Koets

**Affiliations:** a Department of Bacteriology, Host Pathogen Interaction and Diagnostics Development, Wageningen Bioveterinary Research (WBVR), Wageningen University & Research, 8221 RA Lelystad, the Netherlands; b Netherlands Food and Consumer Product Safety Authority (NVWA), P.O. Box 43006, 3540 AA Utrecht, the Netherlands; c Department of Biomolecular Health Sciences, Faculty of Veterinary Medicine, Utrecht University, Utrecht, the Netherlands; d National Tuberculosis Reference Laboratory, Centre for Infectious Disease Control, National Institute for Public Health and the Environment (RIVM), 3721 BA Bilthoven, the Netherlands; e Municipal Health Service (GGD), Department of TB control, 3706 BR Utrecht, the Netherlands; f Municipal Health Service (GGD), Department of TB control, 7200 AA Zutphen, the Netherlands

**Keywords:** Feline tuberculosis, *Mycobacterium bovis*, Zoonosis, Human transmission, Officially bTB free (OTF) country, The Netherlands

## Abstract

Although the Netherlands is an officially bovine tuberculosis (bTB) free (OTF) country, sporadic infections with *Mycobacterium bovis* still cause tuberculosis (TB) in (non-bovine) mammals, including humans. We describe for the first time cases of *M. bovis* infection in domestic cats in the Netherlands with transmission between companion animals and humans.

In January 2023, a domestic cat, euthanized due to severe respiratory clinical signs, was diagnosed with *M. bovis*. Subsequently, three other cats from the household were euthanized and also diagnosed with *M. bovis*. The remaining kitten and dog received antibiotic treatment. Human contacts were screened using Tuberculin Skin Test (TST) and Interferon-Gamma Release Assay (IGRA). Lung lesions were detected in a TST^+^/IGRA^−^ contact which tested positive for *M. bovis* DNA. This human lung-derived *M. bovis* DNA contained single nucleotide polymorphisms (SNPs) that were also identified in the DNA of *M. bovis* isolated from the cats in this household, strengthening the hypothesis of intra-species *M. bovis* transmission within the household. The four TST^+^ human contacts received antibiotic treatment. In the same period, another domestic cat from an unrelated household was euthanized due to respiratory clinical signs and diagnosed with *M. bovis*. This *M. bovis* strain differed 500 SNPs from the strains of the first household and was therefore genetically distinct. Commercially available, ready-to-use raw pet food was a suspected source in both households, however this could not be confirmed.

These cases illustrate the need for one-health vigilance among both veterinarians and human physicians as essential to control outbreaks and prevent further spread to humans, companion animals, wildlife and livestock.

## 1 Background

*Mycobacterium bovis* is the causative agent of bovine tuberculosis (bTB). In several countries, bTB is endemic resulting not only in reduced animal welfare, but also in large economic losses. *M. bovis* has a broad host range not only infecting cattle, but also other mammals, including humans.

In the EU, in accordance with the Implementing Regulation (EU) 2018/1882 of the new Animal Health Law (Regulation (EU) 2016/429), *M. bovis* infection of bovines is a category B disease and requires control/surveillance programs aiming to eradicate the disease. However, for dogs and cats it is a category E disease where control is limited to monitoring. The Netherlands is officially bTB free (OTF) since 1999, indicating that since 1999 consistently <0.1 % of Dutch cattle herds have had a bTB case. Within the OTF status, *M. bovis* infection in cattle may still occur [1]. Besides cattle, other livestock, zoo animals, wildlife and companion animals are also permissive for bTB [2]. Wildlife such as deer, possums, badgers and wild boar may act as a reservoir fueling bTB positive herds [3,4].

*M. bovis* infections in companion animals are classically associated with exposure to *M. bovis* infected cattle. Though other routes of infection are described for *M. bovis* infection in cats and dogs. This includes consumption of contaminated meat [5]. Raw pet food is considered a potential source as a recent large *M. bovis* outbreak in cats associated infection with consumption of commercially available raw pet food [6]. Contact with *M. bovis* infected wild-life is also a potential route of infection [7,8]. Definitive proof of the source remains difficult and therefore potential *M. bovis* source is most often based on an association.

*M. bovis* transmission between pets and humans is sparsely described. Potential intra-species transmission between cats and humans has been detected in a family in the USA [9]. Cat-to-human transmission is described [10] and microbiologically and genetically proven for a case in the UK [11]. This underlines the zoonotic risk of TB in pets. However, *M. bovis* reverse zoonosis; human-to-pet transmission, may also occur [5,12].

In cats cutaneous TB is the most frequently observed form of TB, followed by TB infection of the respiratory and gastrointestinal tract. In cutaneous TB, non-healing skin lesions can be observed showing ulceration. Draining lymph nodes may become infected resulting in lymphadenopathy. *M. bovis* infection can spread to or infect lungs, resulting in weight loss and respiratory clinical signs such as coughing and dyspnea [13].

Here, we describe *M. bovis* infections in cats in an OTF country. The cats resided in two unrelated households and displayed severe clinical signs. For one household, human exposure to the *M. bovis* strain infecting the cats was proven by both indirect diagnostics (Mantoux, interferon-gamma-release assay (IGRA)) and direct detection of *M. bovis* DNA in a lung biopsy. The source(s) of the *M. bovis* infection in the cats could not be identified. This case report is therefore a warning that, even in OTF countries, *M. bovis* infection can occur via yet undefined transmission pathways and that a risk for human exposure remains.

## 2 Case presentation

### 2.1 Case I

**Fig. 1:**
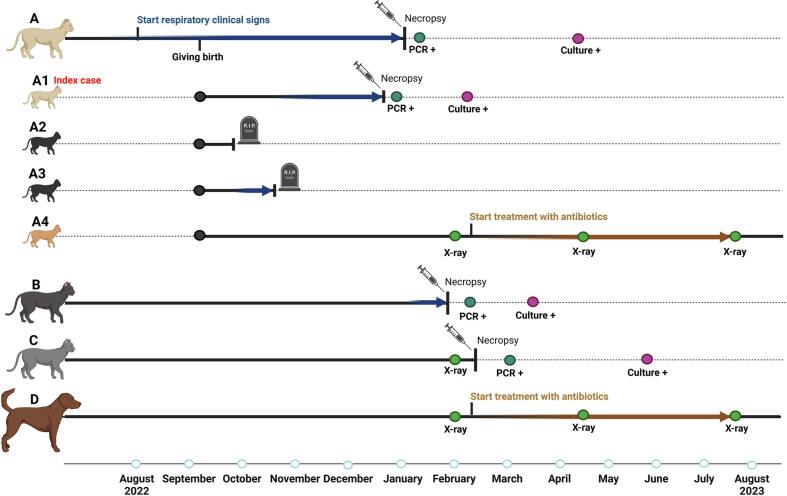
*Timeline Case 1.* Within the household the *index case I* (kitten A1) was one out of four kittens (kitten A1–4) from a litter of *cat A*. *Cat B*, *cat C* and *dog D* also lived in the same household. The onset of clinical signs is indicated with a blue arrow. Time of necropsy, X-ray, PCR and culture results are indicated on the timeline. Prophylactic antibiotic treatment is indicated with a brown arrow. Created in BioRender. https://BioRender.com/k47q463. (For interpretation of the references to colour in this figure legend, the reader is referred to the web version of this article.)

In December 2022, an euthanized 3-month-old Ragdoll kitten (*kitten A1*) was submitted to the Pathology division of the Faculty of Veterinary Medicine, Utrecht University, the Netherlands. The kitten had suffered from dyspnea and was the third kitten from a litter of four that did not survive; two other kittens had already died, one of which had respiratory clinical signs (*kitten litter A1–4*) (Fig. 1). On necropsy, a multifocal granulomatous pneumonia was diagnosed together with similar granulomas in the draining tracheobronchial lymph nodes. In addition, granulomas were seen in the mandibular, retropharyngeal and mesenteric lymph nodes and in the liver. A Ziehl-Neelsen stain of the lung and lymph nodes revealed acid-fast bacilli (AFB) and lymph node and lung tissue were sent to the national reference lab (NRL) for bovine tuberculosis (bTB) (Wageningen Bioveterinary Research, Wageningen University and Research, the Netherlands). IS*6110* PCR and bacteriological culture were performed on the tissue received which tested positive for *Mycobacterium tuberculosis* complex (**Supplemental Table 1**). Colony growth was readily detected in the first month on Löwenstein–Jensen (LJ) medium supplemented with pyruvate. Differential PCR based on presence or absence of specific regions of difference (RD) [14,15] classified *M. bovis* as the causative agent (**Supplemental Table 1**).

**Fig. 2:**
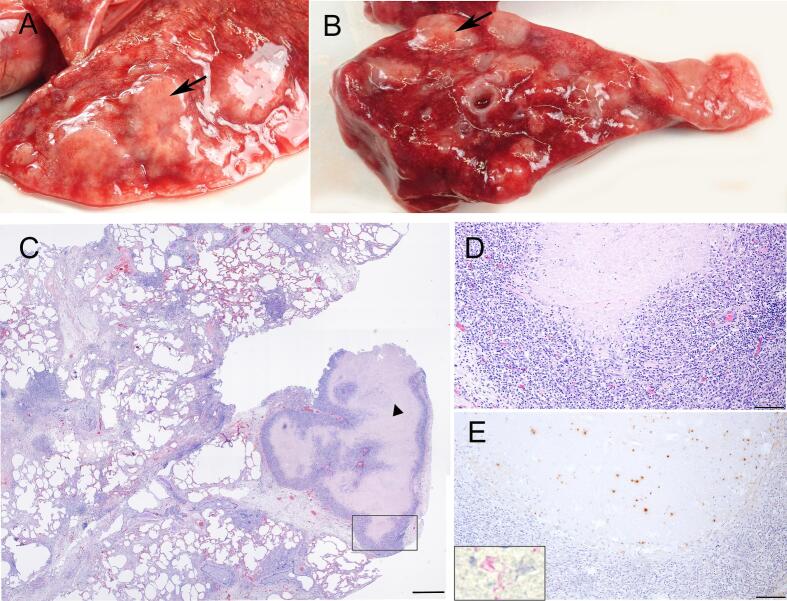
*Lung tissue of cat A.* Multiple granulomas (arrows) are visible on the surface (A) and in the cross section of the lung (B). Granulomas are classified as stage IV granulomas consisting of a confluent core of necrotic lung tissue (arrowhead in C: microscopical hematoxylin-eosin (HE) stain overview of the lung). The central necrosis is surrounded by a band of epitheloid cells, lymphocytes and fibroblasts but no multinucleated giant cells. D: high magnification of the framed rectangle in C. Immunohistochemical staining for mycobacteria shows multiple clusters of positively stained (remnants of) bacteria in the necrotic lung tissue (E). Insert in E: Ziehl-Neehlsen staining of acid-fast bacteria in the necrotic lung tissue. Bar = 1000 μm (C) or 100 μm (D and E).

A few days after the index case was diagnosed with *M. bovis*, the mother of the kitten (*cat A*) was euthanized due to persistent respiratory problems since August 2022 that did not improve. Necropsy at the NRL revealed a granulomatous pneumonia (Fig. 2), granulomatous ileitis and a severely enlarged ileo-caecal lymph node.

**Fig. 3:**
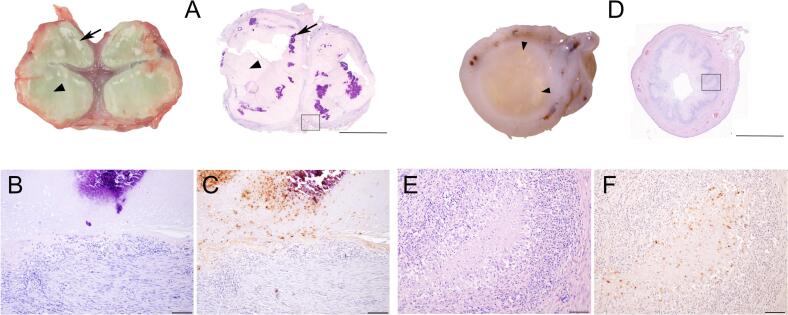
*Intestinal lymph nodes and small intestines of cat A.* Cut surface (A. left) and microscopical HE stained overview (*A. right*) of the enlarged ileo-caecal lymph node which is composed almost entirely of necrotic tissue (arrow heads) with foci of mineralization (arrows).The necrotic tissue is surrounded by a thick fibrous capsule and some epitheloid cells and lymphocytes (B: high magnification of framed rectangle in A). Positive immunohistochemical staining for mycobacteria in serial section (C). Cross section (D. left) and microscopical HE stained overview of the ileum (D. right) showing multiple granulomas in the mucosa and submucosa (arrowheads). Granulomas consist of a central necrotic core surrounded by numerous epitheloid cells, lymphocytes and fibroblasts (E: high magnification of framed rectangle in D.) Positive immunohistochemical staining for mycobacteria in serial section (F). Bar = 5000 μm (A), 2500 μm (D) or 100 μm (B, C, E, F).

On cut surface the ileo-caecal lymph node consisted almost entirely of caseous necrosis with dispersed white foci of calcification surrounded by a thick fibrous capsule (Fig. 3)*.*

In January 2023, another 13-year-old cat from the same household that suffered from coughing and dyspnea (*cat B*) was euthanized and submitted for necropsy. Grossly, a granulomatous pneumonia was seen but there were no macroscopic alterations identified in the small intestines or mesenteric lymph nodes. Both adult cats were IS*6110* PCR positive, differential PCR positive for *M. bovis* and culture positive on tested lymph nodes and lung tissue. *M. bovis* was also cultured from the mesenteric lymph nodes of *cat B*, despite absence of macroscopic lesions. Furthermore, bronchus swabs of *cat A* were negative for *M. bovis* culture whereas bronchial swabs taken from *cat B* were culture positive for *M. bovis*. This indicates the presence of viable bacilli in the airways, suggesting potential open tuberculosis (TB) and ability to spread.

At this time, an adult cat (*cat C*), the last surviving kitten (*kitten A4*) from the litter and a dog (*dog D*) were still part of the household and did not show any clinical signs. In February 2023, an X-ray was performed on all three animals. Although *cat C* appeared healthy, without clinical signs, lung abnormalities were detected on X-ray images and the cat was euthanized. Necropsy of this animal showed a granulomatous pneumonia, granulomatous ileitis and typhlitis, and a granulomatous lymphadenitis of the mesenteric and ileo-caecal lymph nodes. Lymph nodes and lung tissue also tested positive by culture, IS*6110* PCR and differential PCR resulting in *M. bovis*, whereas bronchus swabs were negative for *M. bovis* culture.

On histopathology, as illustrated by Fig. 2 and Fig. 3, the granulomas could be classified up to stage IV with caseous necrosis, calcification and encapsulation [16]. No multinucleated giant cells were seen in any of the granulomas. The Ziehl-Neelsen stain showed a varying number of AFB within the granulomas. Additional immunohistochemical staining using anti-*M. bovis* BCG polyclonal antibodies further validated mycobacteria in the tissue.

The remaining kitten and dog received a prophylactic 5-month antibiotic therapy (azithromycin, rifampicin, marbofloxacine) with follow-up X-rays. Both the kitten and dog finished the treatment and remained in good health. No lung abnormalities were detected on X-ray taken after treatment.

### 2.2 Case II

In February 2023, a 7-month-old castrated male Bengal cat was brought to a referral clinic with severe respiratory clinical signs resulting in extreme shortness of breath. A computed tomography (CT) scan showed a pneumothorax with consolidation of lung tissue and a large gas filled defect in the left caudal lung. Due to the extreme dyspnea, consolidation of lung tissue and AFB-positive lung material, the cat was euthanized and sent in to the Pathology division of the Faculty of Veterinary Medicine, Utrecht University. Necropsy revealed a pneumothorax and granulomatous pneumonia with a cavernous granuloma in the left caudal lung lobe perforating the pleural cavity. In the ileo-caecal lymph node a large granuloma was present with foci of mineralization. Histologically AFB were detected in the lung and ileo-caecal lymph node and thus lung tissue was dispatched to the NRL for further examination on TB. This lung tissue tested positive for *M. bovis* using culture, IS*6110* PCR and differential PCR.

**Fig. 4:**
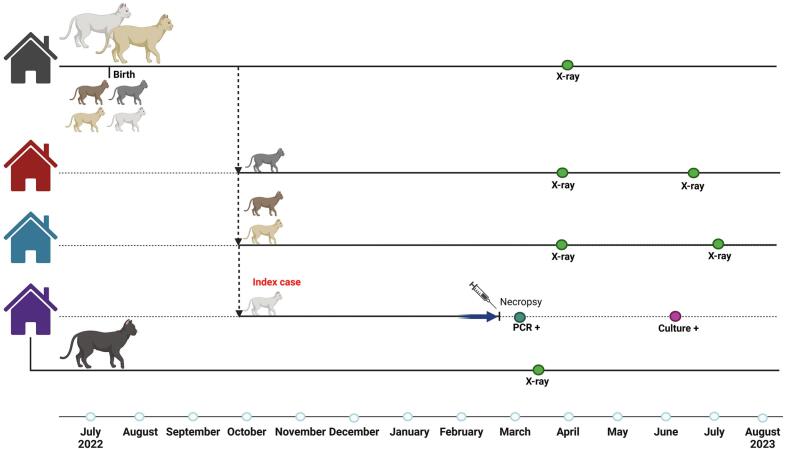
*Timeline Case 1I.* Index case II was one out of four kittens from a litter. The four kittens were distributed over three households. The index case lived with another cat in the new household. The onset of clinical signs is indicated with a blue arrow. Time of necropsy, X-ray, PCR and culture are indicated on the timeline. Created in BioRender. https://BioRender.com/t08m346 (For interpretation of the references to colour in this figure legend, the reader is referred to the web version of this article.)

The index case and his littermates were born in a cattery in July 2022 and were distributed over three different households in October 2022 (Fig. 4). At that time the index case did not show any clinical signs of the respiratory tract, these only developed by the end of January 2023. In March 2023, clinical examinations and X-rays were performed on the parents and littermates of the index case and of a second cat living in same household as the index case. Based on these tests, none of the cats examined were suspected of having an infection with *M. bovis.*

### 2.3 Sequencing

As the two described index cases occurred during the same time period, we hypothesized the potential of a common source. DNA from *M. bovis* isolates from *cat A, index case I (kitten A1), cat B, cat C,* and *index case II* was extracted and used for Illumina sequencing.

Adapter filtering was performed using Trimmomatic (version 0.39) with a sliding window of 5:20, after which read quality was assessed using fastQC (v0.11.9) and visualized with multiqc (version 1.13.dev0). Single nucleotide polymorphism (SNP) calling against the *Mycobacterium tuberculosis* H37RV (NC_000962.3) reference was done using Snippy (4.6.0), while masking repeat regions (PE/PPE/PGRS) using the Mtb_NC_000962.3_mask.bed file as implemented in Snippy).

**Fig. 5:**
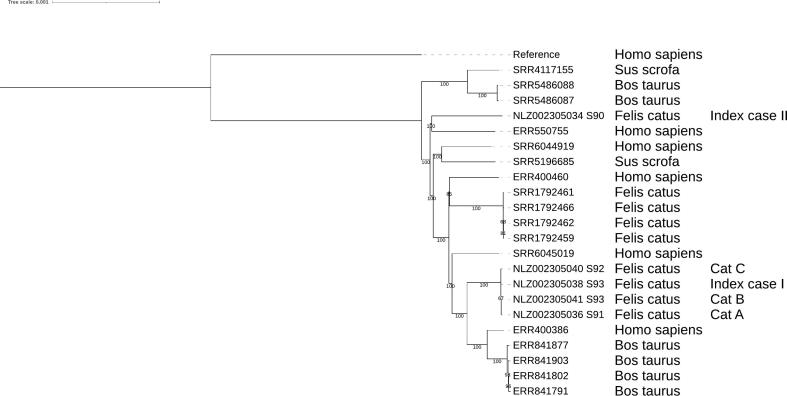
*SNP based phylogenetic tree of M. bovis isolates.* The sequences from the first household cluster together, while *index case II* falls in a separate cluster. *M. bovis* strain names are indicated in the first column. The host of *M. bovis* isolation is shown in the second column. Isolates from cats in this case report are indicated in the third column. The tree is midpoint-rooted, using *M. tuberculosis* H37Rv as outgroup. Numbers on tree nodes represent bootstrap values, the horizontal scale bar indicates nucleotide substitutions per site.

All sequences from the first household cluster together, although one isolate differed by a single SNP, these isolates classified as spoligotype SB0673. The *M. bovis* isolate from *index case II* deviate from the first household with 500 SNPs and classified as SB0121, indicative of an independent infection. The clusters are thus unrelated to each other with respect to the infecting strain (Fig. 5). (data available NCBI: PRJNA1248233).

### 2.4 Human exposure

*Mycobacterium bovis* is zoonotic and therefore human contacts were also tested for *M. bovis* exposure. *Case I.* Tuberculin skin tests (TST) were performed on six individuals who had had (repeated) close contacts with *cat A, kitten A1*, *cat B* and *cat C*. Four contacts tested TST positive (TST^+^) and were subsequently tested for Interferon-Gamma Release Assay (IGRA). Three contacts tested positive (IGRA^+^), indicating exposure to mycobacteria from the tuberculosis complex. In parallel, thorax X-rays were taken of all four TST^+^ contacts. No abnormalities were detected in the TST^+^/IGRA^+^ individuals, however abnormalities were detected in the TST^+^/IGRA^−^ contact. A computed tomography (CT) scan revealed lung lesions in this TST^+^/IGRA^−^ contact and a lung biopsy was collected. No *M. bovis* could be cultured from the material, however, IS*6110* PCR of the tissue tested weakly positive with a Ct >35.

Subsequently, DNA was isolated from the biopsy and analyzed by Hain LineProbe ID (Bruker, DE) and was found to be weakly positive to *M. bovis*. Additionally a *pncA* PCR was performed on the biopsy and by Sanger sequencing the presence of the *M. bovis* specific H57D mutation was confirmed (**Supplementary Table 1**).

Finally, two *M. bovis* regions were selected containing two SNPs that in our database were specific and could differentiate between the *M. bovis* strains isolated from *index cases I* and *II*. The first SNP is located in *mb0151* 172,901 A > C and the second SNP is located in *mb2085* 2,315,608C > T. Primers were selected to amplify the regions containing these SNPs from the biopsy DNA. After Sanger sequencing of the PCR-amplified biopsy DNA, the two specific SNPs in *mb0151* and *mb2085* were identified. The identified SNPs correlate to the SNPs present in the *M. bovis* isolates of the cats from the first household, which are not present in the *M. bovis* isolate from *index case II*. This finding strongly supports the assumption of *M. bovis* transmission within the household between companion animals and humans.

Positive human contacts received prophylactic anti-TB treatment (3 months rifampicin and isoniazid). One positive contact received isoniazid for six weeks, with 6-monthy X-ray follow up for 2 years.

*Case II.* Two individuals had frequent, close contacts with Index case II. TST results were negative for these contacts and thus no exposure or infection was identified in the tested human contacts.

### 2.5 Source tracing

The domestic cats, born in the Netherlands, lived indoors and therefore exposure to (*M. bovis* positive) wildlife, including infected rodents, was considered unlikely. Although rare, humans can also transmit *M. bovis* e.g. [[17], [18], [19], [20]], however we considered this unlikely as the contacts did not present with open TB. Two out of three infected adult cats from the first household, and *index case II* showed macroscopic lesions in the intestine and intestinal lymph nodes. Furthermore, *M. bovis* could be cultured from mesenteric lymph nodes of all euthanized cats from the first household, including *index case A1* and *cat B* that lacked macroscopic lesions. This may indicate that the intestine was the primary route of entry. Upon questionnaire, both households provided commercially purchased raw meat petfood to their (adult) companion animals. The raw meat petfood was typically a 300–500 g sausage containing a mix of raw meat and various organs. Samples of raw meat products that were available at the time of source tracing were collected from the households where *index case I* and *II* were kept, including the cattery in which *index case II* was born. Importantly, the meat products collected came from different batches than the meat products eaten by the *M. bovis* infected cats, as there were no leftover products from these batches available. Samples were taken from different parts of the sausages and processed for PCR, culture and histology. No growth of mycobacterial species was identified and this was in line with negative IS*6110* PCR results. For production of raw meat petfood, among others, organs and tissue from cattle and deer from OTF and non-OTF countries are used.

## 3 Discussion and conclusions

This report describes two concurrent but independent *M. bovis* infections in cats, which involved other potential hosts - a dog and human contacts were exposed. In the first household, in retrospect, clinical signs were already present for over 6 months. This household lost six cats of which four had an *M. bovis* infection confirmed, whereas the remaining kitten, dog and human family members were treated with antibiotics. In the second case description, the infection was limited to one cat. Based on WGS sequencing data, the isolates from the two case descriptions were unrelated and thus infections most likely originated from independent sources. Raw pet food was a suspected source in both households, however this could not be confirmed.

In this case report it was clear that the cats with clinical signs were infected with *M. bovis* as this could be detected post-mortem by direct tests such as the Ziehl-Neelsen histochemical stain, culture and PCR. Unfortunately, detection of latent bTB infection in living companion animals is hampered by the lack of validated indirect diagnostic tests such as feline- or canine-specific TST or IGRA tests [21]. Therefore exposure can only be confirmed using direct tests (PCR, culture) on clinical samples (e.g. pleural fluid of biopsies) or after post-mortem examination. Intriguingly, no multinucleated giant cells were detected in analyzed granulomas. Multinucleated giants cells are characteristic for tuberculosis granulomas [[22], [23], [24]]. However we and also others noticed the lack of these cells in *Mycobacterium bovis* infected cats [[25], [26], [27]].

The Netherlands is an officially bTB free (OTF) country and therefore companion animals are less likely to be exposed to *M. bovis* from either livestock or wildlife [28]. Recently, a large cluster of domestic cats was diagnosed with intestinal bTB that was associated with consumption of raw meat in England and Scotland [6]. The cluster included a total of 47 cats with typical bTB lesions, which tested positive either by IGRA, PCR and/or culture. Infection was confirmed to be caused by *M. bovis* genotype 10:a (SB0272) [29,30]. All cats consumed the same brand of raw venison pet food, however the association was not validated. Based on macroscopic lesions and positive culture of mesenteric lymph nodes in our cases, we hypothesize that *M. bovis* could be introduced to the intestine of the adult cats by consumption of *M. bovis* contaminated petfood. Cats from both households were fed commercial raw petfood, but no *M. bovis* could be identified in the batches available for testing. Although the involvement of the mesenteric and the ileo-caecal lymph nodes and the presence of a granulomatous ileitis are indicative for infection by ingestion of infected material (feed or milk), other transmission routes cannot be excluded as, for example, contaminated sputum can be ingested. Furthermore, due to close contacts in the first case description, transmission via aerosols must be taken into account. The isolation of *M. bovis* from bronchial swabs of *cat B* confirmed the presence of viable bacilli in the respiratory tract and this emphasizes the risk of aerosol transmission. This can also explain the identification of positive human contacts, of which one case was validated to be infected with *M. bovis*.

Feline intestinal bTB, due to consumption of infected raw dairy products, occurred often before the implementation of national TB control programs [31]. Most described clinical manifestations of feline TB include cutaneous lesions, which could be ascribed to direct contact to infected wildlife [8]. However, as described in the latter, raw pet food could also be a potential risk. Transmission of other diseases via raw pet food is also reported, including *Brucella suis* infection in a dog after consumption of commercially available mixed raw feed [32], but also other pathogens as summarized in [33]. The composition of the meat in our case was characterized and comprised mostly of muscle and fat. In addition, the following tissues could be identified; tripe, kidney, liver, cartilage, bone and lung tissue (data not shown). Based on our these results and some general ingredient lists of raw pet food providers, it is clear that organs such as lungs are present in raw pet food. As these organs are also derived from non-OTF countries, it is likely that these may contain *M. bovis,* potentially leading to morbidity and mortality among companion animals and eventually humans.

Abattoirs have strict regimens regarding food safety. Veterinarians perform post-mortem inspection of carcasses to screen for abnormalities. However, these screens are based on macroscopic inspection and thus, in the case of bTB, can miss an infected animal yet to develop visible lesions but still harboring viable bacilli [34]. Such infected animals have been described before as non-visible-lesion (NVL) bTB [35]. NVL are described for TST^+^ cattle. TST^+^NVL animals can be false positive due to cross-reactivity against conserved antigens present in related (environmental) bacteria [36]. However, *M. bovis* can still be detected in either TST^+^NVL and even TST^−^NVL animals [37] indicating that macroscopic post-mortem inspection is not sufficient to detect all *M. bovis* positive animals at *s*laughterhouses. Thus lack of sensitivity and specificity in both ante-mortem and post-mortem diagnostics hampers *M. bovis* detection. Clinical signs may appear from days to even years after infection [38,39]. Thus carcasses and meat that pass inspection might still contain viable bacilli and potentially enter the raw pet food chain. It is possible that a contaminated batch of raw pet food was consumed months or years before onset of the first clinical signs. Therefore, it is not surprising that it is usually not possible to obtain a sample from the infected food source at the time the infection is detected.

To the best of our knowledge this is the first report describing a feline bTB case in an OTF country. Previous described cases occurred in bTB endemic regions or countries, including UK [6,25], Ukraine [40,41], Turkey [42] and USA [9]. Potential routes of infection ranged from contact with an infected badger [25], oral exposure [6,42], infection during surgery [40] or a bite from another animal [41]. Importantly, most of these cases describe additional TB cases in animal [25,41] or human [6,11] contacts, as we also describe in this case report. Infections may occur from the same initial source in the household or due to subsequent transmission. O'Connor et al. showed that transmission of *M. bovis* from cat to human is indeed possible as a human case developed bTB after contact with an *M. bovis* positive cat. Both human and cat *M. bovis* isolates were genetically indistinguishable [11]. This has led to a change in the perspective that transmission from cat to human is not neglectable, but is considered a low risk [43]. In our case report, four out of six human contacts in the first household tested positive for TB infection, but did not develop active bTB up to writing this case report. It is unclear what factors contribute to transmission, which could include repeated close contact with infected animals. In addition, it is also unknown how rare these feline bTB cases are, as it is unclear whether and how many of these cases remain undiagnosed or misdiagnosed.

In general, guidelines on how to act in the event of bTB in non-bovines, including companion animals, are lacking and may impact bTB control. In this case report a kitten and dog were treated prophylactically with a combination of antibiotics. In Italy, a non-OTF country, several *M. bovis* infected cats were treated with marbofloxacin, doxycycline and azithromycin, but after 5 weeks they were euthanized based on Italian law on *M. bovis* identification [41]. Another case in Texas described a successful treatment (up to 21 months after start) of an active feline bTB and asymptomatic bTB cat using rifampin, marbofloxacin, and clarithromycin [9]. Gunn-Moore summarized the outcome of mycobacterial treatment in 184 cats, including infection with *M. bovis*, *Mycobacterium microti* and *Mycobacterium avium*. Treatment ranged from monotherapy to triple therapy and treatment duration of less than one month to more than 6 months. Although complete remission is described, over 60 % of cases showed relapse [26]. Relapse of infection is not only confined to animal TB, but is also well-described for human TB cases [44]. In Europe, bTB in cattle is a notifiable disease and defined as category B, meaning that eradication measures are obliged (the Implementing Regulation (EU) 2018/1882 of the new Animal Health Law (Regulation (EU) 2016/429), including isolation and culling, whereas treatment is strictly forbidden. Nevertheless, (multi)drug-resistant *M. bovis* strains exist [[45], [46], [47]], which might be introduced by treatment of *M. bovis* infected humans. Together with the development of drug resistance, the relapse of infection sets the stage for the complexity of successfully treating TB cases, with the risk of reactivation of the disease leading to possible further spread among animals and humans.

Overall, TB should be considered as a differential diagnosis upon presentation of respiratory signs in companion animals. As we detected *M. bovis* in an OTF country, we should strengthen and continue bTB control and limit potential spread in order to maintain the bTB free status. We were not able to positively identify a source of infection for either household. Undetected *M. bovis* infections in free roaming companion animals are also a potential risk for livestock as well as wildlife. Lack of control may lead to further spread of infection and regaining control can take many years as has been described for France [48].

With this case report we would like to stress that sporadic cases of bTB in mammals can still occur in an OTF country. Furthermore, the cases described illustrate the need for one-health vigilance among both veterinarians and human physicians as essential to control outbreaks and prevent further spread to humans, companion animals, wildlife and livestock.

## CRediT authorship contribution statement

**Susanna Commandeur:** Writing – review & editing, Writing – original draft, Investigation, Conceptualization. **Marleen van der Most:** Writing – review & editing, Writing – original draft, Investigation, Conceptualization. **Jeroen Koomen:** Writing – review & editing, Formal analysis. **Lucien van Keulen:** Writing – review & editing, Investigation. **Annemieke Dinkla:** Writing – review & editing, Investigation. **Xander Luinenburg:** Writing – review & editing, Investigation. **Marieke Escher:** Writing – review & editing, Investigation. **Pieter Jacobs:** Writing – review & editing, Investigation. **Ingrid Keur:** Writing – review & editing, Investigation. **Guy C.M. Grinwis:** Writing – review & editing, Investigation. **Erik Weerts:** Writing – review & editing, Investigation. **Els M. Broens:** Writing – review & editing, Investigation. **Richard Anthony:** Writing – review & editing, Investigation, Formal analysis, Data curation. **Miranda Kamst-van Agterveld:** Writing – review & editing, Investigation. **Karin Rebel:** Writing – review & editing, Investigation. **Erik Huisman:** Writing – review & editing, Investigation. **Marloes Heijne:** Writing – review & editing, Conceptualization. **Ad Koets:** Writing – review & editing, Conceptualization.

## Consent

Oral informed consent was provided from household members to analyze and present the data obtained from both companion animals and human contacts.

## Funding sources

This work was funded by Ministry of Agriculture, Fisheries, Food Security and Nature through the project of the NRL bovine TB, under grant number WOT-01-002-002.02.

## Declaration of competing interest

The authors declare that they have no competing interest.

## Data Availability

data available NCBI: PRJNA1248233

## Appendix A Supplementary data

Supplementary material Image 1
